# Rearrangement of Mentoring Components for Student Achievement of Medical Universities

**DOI:** 10.30476/JAMP.2022.93404.1526

**Published:** 2022-07-01

**Authors:** AHMAD KEYKHA, ELHAM KEYKHA

**Affiliations:** 1 Department of Educational Management and Planning, Faculty of Psychology and Educational Sciences, University of Tehran, Tehran, Iran; 2 Department of Oral Medicine, Dentistry School, Qom University of Medical Science and Health Services, Qom, Iran

**Keywords:** Mentoring, Medical students, Counseling, Preceptorship, Achievement

## Abstract

**Introduction:**

Mentoring programs are the most important factor in the achievement of students' human capital. However, in Iran's higher health education system, these initiatives have received less attention. The goal of this research is to reorganize the components of mentoring for medical university student achievement.

**Methods:**

This qualitative study was conducted using a Meta synthesis method. Keywords of mentoring medical students, mentoring academics students, human capital development, student development, and mentoring were searched in database: Science Direct, Springer, Wiley Online Library, ERIC, Sage, Emerald, Pub Med from 2000 to 2021. Out of total 91 studies, finally 51 studies were selected.

**Results:**

The findings are divided into two parts. The first part deals with the characteristics of faculty members and students in the mentoring programs of medical universities. These features include student-specific characteristics, faculty-specific characteristics and common characteristics. In the second part of findings, the components of mentoring programs were extracted. These components include university, communication, soft capacities; before the implementation of the program, during program implementation, monitoring and evaluation of program implementation, and consequences of program implementation.

**Conclusion:**

The suggested components should be examined by managers of the higher health education system for student human capital development due to the relevance of mentoring programs in the development of medical students' human capital.

## Introduction

Mentoring programs are used as a main tool in higher education to increase students' personal and professional growth ( 1 ). In the higher health education system, mentoring programs have been constantly evolving and developing since the 1970s. But, they have been officially introduced in medical education since the late 1990s. However, in most countries there is deficiency for mentoring programs for medical students ( 2 ). Despite the paucity of mentorship programs in the higher education system, most studies believe that these programs give an excellent chance for the development of human capital ( 3 , 4 ). Research findings confirm that the implementation of mentoring programs has positive results in developing the students of all levels of medicine, nursing, etc. ( 5 - 7 ), especially in the field of training program for talented students who could replace the specialized faculty members in the future ( 8 ). These programs are recognized as being critical to students' success in medicine ( 9 , 10 ). This limited number of mentoring program installations has resulted in some failures. The ambiguous nature and boundaries of the mentee-mentor relationship, as well as a lack of mutual trust, are among the causes for failure in Christie's research. Gus, et al. found other factors for failure of mentoring programs, such as inappropriate relationships, lack of compatibility, poor personality and impersonality between mentee and mentor, poor feedback, poor support ( 11).

In general, regarding the importance and role of mentoring programs in student achievement, few studies were done in the field of health system mentoring. Another noteworthy point is the lack of attention to this issue in the domestic research literature. For this reason, Meta synthesis and synthesis method of studies conducted in this field in the world were used. In our study, in addition to presenting a comprehensive and deeper view of this subject, an attempt was made to collect many of causes and factors of program failure according to a systematic and comprehensive method. The purpose of this study is the rearrangement of mentoring components for the achievement of students in medical universities. 

Historically, the word "mentor" originated in the mid-eighteenth century, in the epic of Homer and Odyssey. The name is derived from the name of a friend who Odysseus entrusted his son to him as a trusted advisor ( 12 ). Nevertheless, there is still no universal definition of mentoring among scholars despite the fact that the number of articles in the field of mentoring at the university has grown significantly over the last five years ( 13 ). According to a study by Karuna et al., Mentoring is a process in which a more experienced person guides another person (usually younger) to lead to learning, personal growth, and professional development ( 14 ). Peake and Kelly believe that mentoring is a multi-dimensional concept related to evaluation, monitoring, forecasting, and guidance. All of these elements must be present in a mentoring relationship ( 15 ). Mentoring, according to Walker-Reed, is a kind of learning assistance that aims to adjust a trainee to new conditions via professional changes ( 16 ). In general, mentoring programs run in five modes; 1) group mentoring; 2) one mentor and one mentee; 3) one mentor and several mentees; 4) several mentors and several mentees; and 5) several mentors of one mentee ( 17 ). If any of the mentorship program techniques are applied, it will have considerable good benefits and repercussions for medical students, including academic performance, improved research quality, professional growth, improved student welfare, and development of personal identity ( 18 - 23 ). In terms of theoretical implications, the three theories can be generalized to mentoring medical students. 1) Self-determination theory, according to which, humans have mental needs beyond physiological needs such as food and shelter. In particular, human beings need competence, dependence and independence to perform purposeful activities that result in meeting these needs. Intrinsic motivation is the major axis. As a result, students might be encouraged to satisfy their goals by obtaining these abilities through improving skills via mentorship interactions ( 24 ). 2) socialization theory; Sociability is the process by which individuals acquire the attitudes, beliefs, values, and skills necessary to live in a (organizational) society ( 25 ). Through mentoring programs, it is possible for students to adapt to the university and university culture and to recognize unwritten rules and norms to accelerate their scientific socialization. 3) Theory of human capital, which means teaching people to accumulate knowledge and develop skills and capacities for economic value creation. One of the major methods of this theory in the organization (university) is to meet the demands of current human capital and replace skills in order to develop innovation ( 26 ). Student mentorship programs provide the groundwork for identifying and replacing future human capital by nurturing and developing students' present human capital. Hamby et al. examined the experiences of mentees in a medical student mentoring program and found that 84% of mentees were satisfied with the quality of medical students' work and 85% with the quality of the program. Another important point was that 84% of the mentees were interested in participating in the next courses of mentoring medical students ( 27 ). In a qualitative study, Roche et al. examined the experiences of medical students who served as mentors. The relevance of mentorship in medicine via personal counseling and defining future professional objectives was the major emphasis of the interview analysis ( 28 ). Riskin et al. ( 29 ) in a study of group mentoring for medical students found that 91% of educators considered their main motivation for joining this course was to help the personal, social and professional growth of medical students. Boyd et al. ( 30 ) in a study of medical program selection programs for medical students concluded that 81% of school students considered mentoring to be very important for their profession and stated important achievements such as writing skills, statistical analysis, etc. Ng et al. ( 31 ), in a qualitative study, represented the experiences of medical students on the benefits and effects of a mentoring program. Findings revolved around four main areas: identification, integration, feedback, and seniority. Students found mentoring useful for team integration and an opportunity for constructive feedback on their clinical and professional skills. Moreover, this study aims to rearrange the components of mentoring for the advancement of medical students.

## Methods

**Figure 1:**
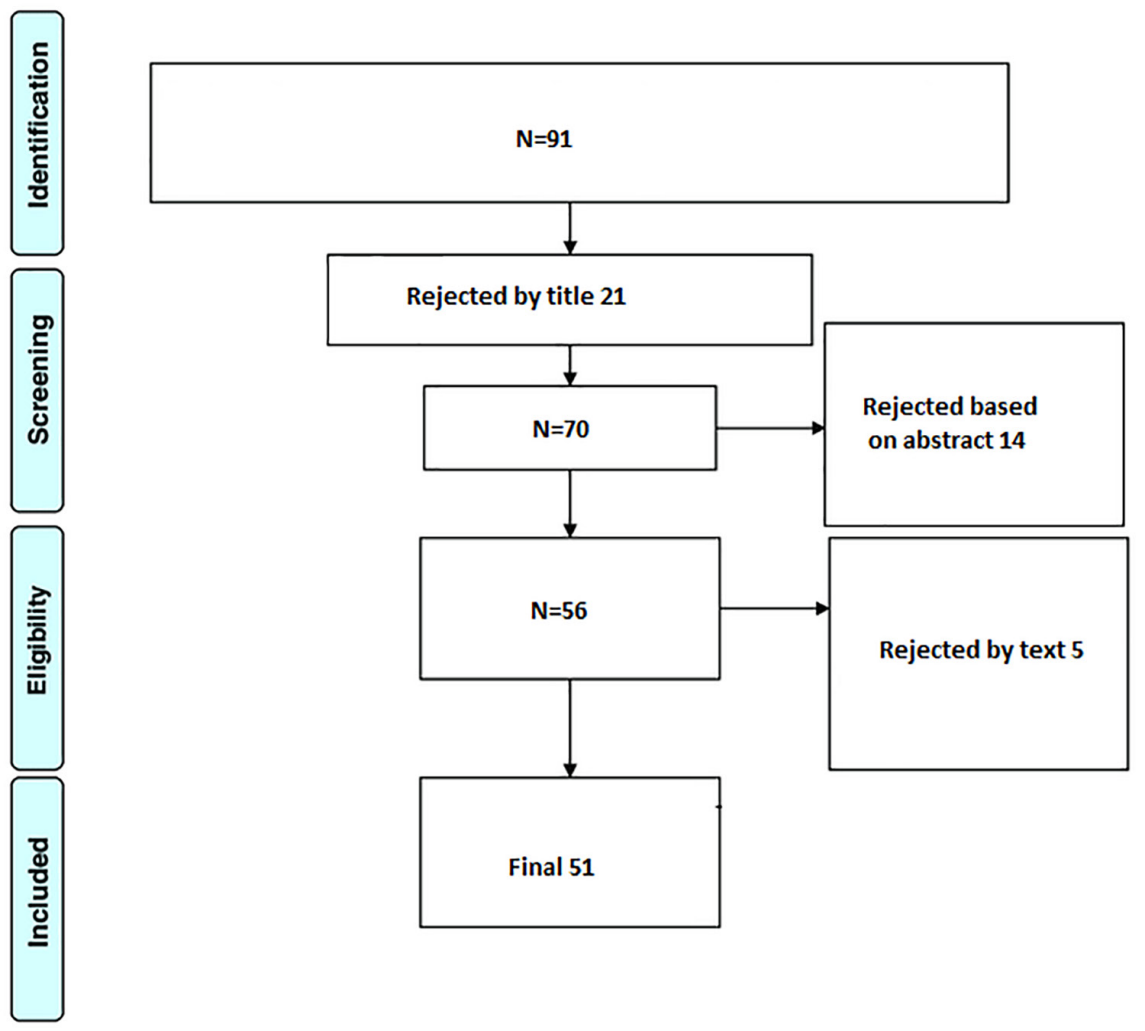
The process of screening searched articles

To review and synthesize the research conducted on the topic, the seven-step Meta-synthesis method was used ( 32 ). The first step is the formulation of research questions. This study is centered on two primary questions: first, the basic features of medical university students and professors, and second, the main components of medical university mentorship programs. The systematic review of the literature is the second phase. For this purpose, specific research terms such as mentoring medical students and mentoring academics students during the period 2000–2021 were searched in the databases Science Direct, Springer, Wiley Online Library, ERIC, Sage, Emerald and Pub med, and a total of 91 studies were found. In the third step, we made sure that the screening and selection of research was appropriate. The articles acquired were examined in multiple phases, with the findings being compared to the study goal. Criteria for article selection included the field of study, type of study (quantitative, qualitative, and mixed), consideration of the desired time frame, and access to the full text of the article. Articles that did not fit the topic according to the PRISMA form were excluded in three stages: Title Review, Abstract Review, and Text Review. Figure 1 describes the process of screening and selecting articles.

The fourth step is to extract information from the articles. As a consequence, the titles, purposes, and summaries of the outcomes of the articles were recorded. The findings were then integrated, combined, and interpreted in the fifth stage. The qualitative content analysis approach was employed in this stage. First, each article's essential themes were extracted independently. The major ideas were then divided into primary and secondary subcomponents based on their similarities and differences. The sixth step was to check the quality of the findings. To validate the findings, the method was reviewed by the research colleague. The seventh step was the provision of results.

### Ethical Consideration

In all stages of the present study, the ethical principle of fidelity was observed in citing sources and using their results.

## Results

To answer the first research question, i.e., what are the most important characteristics of medical students and faculty in the University of Medical Sciences, the terms were categorized based on similarities and differences after extraction. 

**1. Characteristics of faculty; a) general characteristics:** having no judgment and prejudice ( 14 , 33 - 35 ); empathic ( 36 - 38 ); kind and compassionate ( 39 , 40 ); humble ( 39 ); fair ( 41 ); inspiring ( 36 , 42 , 43 ); trustworthy ( 44 ); role model ( 45 , 46 ); up-to-date ( 45 , 46 ); encouraging ( 35 , 43 , 47 ); honest ( 35 , 40 , 48 , 49 ); patient ( 34 , 35 , 37 , 49 , 50 ); supportive ( 36 ); optimistic ( 36 ); altruistic ( 35 ); **b) scientific characteristics:** available ( 51 - 57 ); with specialized knowledge ( 35 , 51 , 54 , 56 , 58 ); continuous facilitator ( 51 , 59 , 60 ); experienced ( 2 , 14 , 35 , 36 , 47 , 54 , 56 , 58 , 60 , 61 ); continuous assessor of student performance ( 33 , 34 , 41 , 43 , 52 ); emotionally supportive ( 38 , 39 , 53 , 62 , 63 ); experienced ( 33 ); identifying and attending to student’s interests and needs ( 33 , 44 , 50 , 54 , 64 , 65 ); career counselor ( 2 , 41 , 42 , 62 , 66 ); personal counselor ( 33 , 38 , 56 , 64 ); vocational and academic counselor ( 60 , 62 , 64 , 67 - 70 ); work supportive ( 41 ); identifying strengths and weaknesses of students ( 71 ).

**2. Characteristics of student; a) general characteristics:** active listener ( 13 , 33 , 40 , 55 , 62 , 72 ); self-reflective ( 45 ); self-critical ( 43 , 45 , 55 ); polite ( 55 ); **b) academic characteristics:** adaptability ( 39 ); active participation in professor’s research ( 73 ); appreciation of professor ( 74 ); accepting weaknesses ( 74 ); setting individual learning goals ( 50 ); analyzing own mistakes ( 49 ).

**3. Common characteristics; a) common general characteristics:** interest (positive attitude) ( 33 , 36 , 39 , 42 , 49 , 51 , 58 , 75 ); voluntary participation ( 2 , 51 , 62 , 67 ); mutual commitment ( 53 , 57 , 60 , 70 , 71 , 74 , 76 , 77 ); acceptance of criticism ( 14 , 33 ); recognition of mutual accountability ( 47 ); mutual responsibility ( 52 ); mutual respect ( 34 , 38 , 40 , 42 , 49 , 50 , 59 ); punctuality ( 33 ); mutual relationship ( 37 , 48 , 50 , 73 ); similar interests ( 2 , 37 , 40 , 49 , 58 , 78 ); discipline ( 44 ); flexibility ( 34 , 44 , 79 ); willingness to work ( 36 , 45 , 74 ); conscientiousness ( 61 ); motivation ( 34 , 60 , 65 ); acceptance of others’ ideas ( 40 ); change in work habits ( 77 ); openness ( 79 ); common tendencies ( 46 ); **b) common academic characteristics:** knowledge sharing ( 47 , 52 , 77 ); clear expectations ( 52 , 72 ); agreed expectations and goals ( 59 ); understanding peer expectations ( 47 , 59 ); clarifying values ( 59 ); safe environment for learning ( 40 , 50 , 59 , 74 , 78 , 80 ); consensus expectations ( 53 ); mutual responsibility ( 35 , 39 , 41 , 46 , 53 , 78 ); mutual commitment for implementing the program ( 53 , 68 , 73 ); sharing experience ( 33 , 77 ); having clear expectations ( 33 , 38 , 46 , 54 , 56 , 63 , 64 ); challenging each other ( 73 ); having realistic expectations ( 55 , 73 ); having clear goals ( 44 , 56 , 58 , 78 ); mutual responsibility ( 45 , 49 , 74 , 78 , 80 ); setting achievable and realistic goals ( 50 , 70 , 74 ); mutual understanding of goals ( 54 , 58 ); sharing ideas ( 40 ); sharing concerns ( 40 ); adapting to the program ( 77 ); engaging students in activities ( 46 ). In fact, these features are critical for both (students and professors) to start a mentoring program. Some features are specialized, but some features are common. Based on research, all these features (specialized and common) were extracted. Having these features is very important for better execution of mentoring program.

In order to answer the second research question, i.e. “what are the main components of medical universities’ mentoring programs”, a categorization was also made after extracting the key terms.

**1. Academic; a) university leadership:** coordination among various shareholders in the university ( 52 ); organizational support ( 52 ); reward system ( 53 ); establishment of formal policies for the program ( 53 ); continuous evaluation of faculty ( 39 , 53 ); inclusion of mentoring programs in student enrollment ( 51 , 63 ); recognition of mentoring programs in university policies ( 14 , 54 , 62 , 67 ); job security ( 53 ); giving appropriate salaries to faculty ( 53 , 73 ); modification of educational plan and curricula ( 33 ); enhancement of educational quality ( 62 ); internationalization of universities ( 62 ); allocation of sufficient financial resources for mentoring programs ( 62 , 73 ); alignment of mentoring goals with university goals ( 63 ); definition of a mentoring program in the university’s mission and vision ( 41 ); improvement of university performance ( 81 ); development of clear policies ( 73 ); ratio of number of professors to students ( 73 ); efficiency of organizational structure ( 58 ); **b) university management:** development of internship programs ( 42 , 47 , 54 , 66 , 76 , 77 , 81 ); adjustment of workload of faculty ( 14 ); development of university facilities ( 52 ); financial support ( 33 , 35 , 52 , 62 , 66 , 73 , 78 ); financial awards ( 53 , 70 ); use of incentives for professors ( 33 , 58 , 62 , 66 , 72 ); conflict management ( 33 , 45 , 49 ); scholarships ( 58 , 66 , 73 ); payment of fees to professors and students ( 73 ); session for the diverse needs of students ( 73 ); use of skills of retired professors; special attention to mentoring program for female medical students ( 42 ); attention to mentoring program for medical students ( 66 ); management strategies ( 44 ); development of the mentoring program as a course ( 58 , 60 ); development of rules in mentoring ( 50 ); allocation of research funds ( 60 ); support of the university president ( 54 ). This component is related to planning design activities within the university. This component includes two high (leadership) and low levels (management) of the university. Importantly, managers at medical universities believe in the importance of student mentoring programs. Therefore, it is suggested that the scope of support for managers be for both students and faculty members. In fact, university policies should be in accordance with the mentoring program. 

**2. Communication; a) development of communication:** relationships based on mutual trust ( 34 , 38 , 45 , 51 , 55 , 59 , 60 , 74 , 79 , 80 ); development of informal interactions ( 41 , 47 , 49 , 59 , 64 , 77 , 78 ); continuous and regular communication ( 14 ); development of written and oral relationships ( 39 , 58 , 59 ); networking relationships ( 33 , 36 , 59 , 60 , 78 ); development of relationships with other faculty ( 40 , 66 , 70 ); development of relationships with other students ( 39 , 61 , 66 , 77 ); development of interactions with university staff ( 61 ); increased interaction with physicians ( 56 ); **b) communication management:** improving the quality of relationships ( 34 , 53 ); encouraging interactions and communication ( 53 ); non-hierarchical relationships ( 53 ); non-competitive relationships ( 53 ); facilitating professor-student relationships ( 33 ); creating lasting relationships ( 72 ); creating a friendly atmosphere ( 72 ); creating a dynamic environment ( 39 ); dynamic relationships ( 78 ); professional relationships ( 49 ). The communication component plays an important role in the more effective implementation of the mentoring program. Because, if the relationship is not bilateral and continuous, the mentoring program cannot be effective. These relationships need to be both developed and managed. Managers and program participants (professors and students) should strive to expand relationships. In fact, everyone as a team in this program should be connected to each other, striving for a single goal.

**3. Soft capacities; a) cultural norms:** pro-mentoring academic culture ( 37 , 44 , 52 , 53 , 65 ); paying attention to professional standards and values ( 41 , 52 ); learning culture ( 52 , 71 ); changing academic values and attitudes ( 53 ); favorable organizational climate ( 53 ); cultural compromise and convergence ( 53 ); creating a shared cultural identity ( 53 ); collective culture ( 37 , 53 ); multicultural ( 33 ); generational differences between students and professors ( 39 , 47 ); eliminating discrimination norms in the university ( 42 ); supportive culture ( 58 ); research culture ( 58 ); recognition of culture ( 48 ); shared values and beliefs ( 37 ); **b) moral norms:** work ethics ( 37 , 53 ); awareness of research ethics ( 53 ); developing ethical behaviors ( 36 , 53 , 79 ); ethical commitments ( 36 ); developing professional ethics ( 72 ); creating ethical values ( 77 ). This component is very important. Because it is the starting point of any cultural changes. University environment and values should support the implementation of the mentoring program. The organizational culture of the university should be consistent with the mentoring programs. In addition, ethical standards must be observed in this program. All members must adhere to moral values.

**4. Prior to program implementation; a) pre-planning:** knowledge and awareness of the benefits of the mentoring program ( 62 ); systematic program design ( 39 ); accurate program information ( 38 , 39 , 64 ); production of mentoring visual and audio content ( 41 , 81 ); writing guidelines for sessions ( 47 , 54 , 64 ); belief in the usefulness of the mentoring program ( 64 ); understanding of the importance and role of mentoring programs ( 73 ); creating competition in the intake of program ( 2 ); student needs assessment ( 60 ); pre-implementation coordination ( 68 ); **b) preparation:** preparation of student; preparation of professor ( 44 , 55 , 73 ); fit of personality of professor to student ( 51 , 73 ); student freedom in selection of professor ( 60 , 76 ); skills training workshops in advance ( 14 ); match between personality of professor and student ( 47 , 59 , 64 , 78 ); selection of professor tailored to students’ needs and interests ( 59 ); development of common agenda ( 74 ). This component is to prepare the mentoring program. This is an important point that must be carefully considered. All stages of the program must be systematically identified. Everyone's roles and expectations should be clearly defined. It is very important to conduct a needs assessment of all members before implementing the program. In addition, the important point is that all members should be involved in the design of the program. 

**5. During the program implementation; a) management of program implementation:** developing leadership directives ( 14 , 57 , 77 , 80 ); defining roles ( 52 , 55 , 78 ); drafting specific and codified policies ( 4 ); team mentoring ( 36 , 56 , 59 , 62 ); interdisciplinary mentoring ( 35 , 59 , 73 ); adopting goal-oriented strategies ( 33 ); leadership directives for the program ( 33 , 43 ); drawing short- and long-term goals ( 56 , 60 , 67 ); prioritizing mentoring program goals ( 62 ); drawing various goals for the mentoring program ( 62 ); drawing a chart time in achieving the goals ( 39 , 47 , 63 , 78 ); customizing the contract for program implementation ( 63 ); customizing the program to meet needs ( 63 ); transparency in program implementation ( 41 , 46 , 78 ); use of specialized staff resources in program implementation ( 73 ); pre-implementation agreement ( 43 ); measuring progress in achieving goals ( 60 ); goal setting tailored to the needs and interests of the student ( 71 ); formation of mentoring committees ( 38 ); **b) how to conduct sessions:** recording and review of sessions by students ( 68 ); holding sessions beyond schedule ( 68 ); time management of sessions ( 37 ); extracurricular activities ( 67 , 76 ); mutual commitment to program implementation ( 14 ); standardization of session modules ( 68 ); organization of sessions ( 14 , 39 ); increasing informal sessions ( 56 , 70 ); group sessions ( 58 , 59 , 64 ); matching content of sessions to goals ( 59 ); active participation of both sides in sessions ( 33 , 75 ); adequate number of sessions ( 62 ); holding regular sessions ( 44 , 54 , 58 , 62 ); increasing number of sessions ( 78 ); keeping total session time ( 71 , 78 ); diversification of communication tools ( 47 , 62 ); documentation of sessions ( 54 , 63 , 68 ); assigning timing of sessions in consultation with both sides ( 63 ); selecting the right venue for sessions ( 38 , 39 , 48 ); agreeing on a venue ( 38 ); designing student-centered activities ( 41 ); increasing attraction of sessions ( 81 ); teaching methods in line with students’ interests and needs ( 81 ); simulating medical skills ( 81 ); precisely assigning the topics of each session ( 75 ); flexibility in implementation ( 60 ); holding weekly sessions ( 74 ); forming small groups in sessions ( 74 ); studying and preparing before teaching sessions ( 45 ); prioritizing topics in sessions ( 58 ); regular feedback-sessions ( 43 , 65 ); continuous reflection during the implementation of the program ( 65 ); use of different mentoring styles ( 65 ); purposeful planning of the sessions ( 49 , 54 ); allocation of sufficient time for the implementation of the program ( 56 ). This component refers to the performance of the program. For this purpose, meetings should be managed regularly. Check the implementation of the program continuously. Pay attention to how the sessions are conducted. Accordingly, the goals of the meetings should be clear and accessible. In addition, the goals must be appropriate to needs. In the implementation of the meetings, the above points should be considered. These items help to perform the program better and increase the efficiency of the program sessions.

**6. Monitoring and evaluating the program implementation; a) performance monitoring:** continuous monitoring ( 46 , 74 , 78 ); monitoring of student performance ( 47 , 52 ); development of supervision rules ( 52 ); effective monitoring of program implementation ( 37 , 43 , 53 , 63 ); **b) program evaluation:** continuous and regular feedback ( 33 , 34 , 47 , 52 , 81 ); continuous evaluation of program gains and losses ( 53 ); attention to mentoring programs among faculty ( 53 ); rewards for success in the program ( 33 ); profiling of each student’s performance information ( 63 ); receiving feedback from program implementation for correction ( 63 ); attention to mentoring programs when promoting faculty academically ( 56 , 64 ); accurately define outcomes of program implementation ( 73 ); continuously evaluate sessions ( 72 ); continuously evaluate programs ( 35 , 41 , 43 , 55 ); evaluate program effectiveness ( 2 , 38 , 68 ); continuously evaluate student progress ( 2 ); provide feedback based on goals set; annual performance reports ( 60 ). This component refers to program monitoring and evaluation, which is an important component of the program. Therefore, the performance of the program must be constantly monitored (self-monitoring by program members and external monitoring). Then the weaknesses must be eliminated and the strengths reinforced. The important point is that monitoring and evaluation should be continuous. Because the results of each program can be used to modify the program and then upgrade the next program. 

**Figure 2:**
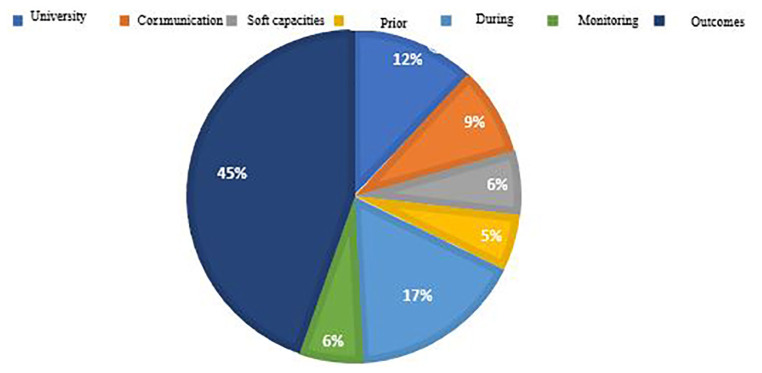
Percentage of components of the student mentoring program of the Medical Sciences Universities.

**7. Outcomes of program implementation; a) outcomes for students:** development of clinical skills ( 34 , 51 , 62 , 69 , 78 ); critical thinking skills ( 39 , 79 , 80 ); stress management ( 36 , 39 , 46 , 48 , 57 , 64 ); reflection on alternative strategies in monitoring ( 51 ); improvement in academic performance ( 51 , 61 , 64 , 70 , 72 ); workload balance ( 36 , 56 , 58 ); application of theory in practice ( 50 - 52 ); practice based learning ( 46 ); career advancement ( 53 , 56 , 60 , 67 , 69 ); personal growth ( 39 , 48 , 59 , 76 , 77 ); improving research capacity ( 54 , 66 , 67 ); improving interpersonal communication skills ( 39 , 67 , 69 , 79 ); development of soft skills ( 76 ); balancing work and life ( 56 , 68 , 69 , 76 ); increasing self-confidence ( 38 , 54 , 61 , 69 , 73 , 80 ); optimizing decision making for continuing education ( 14 ); individual student independence ( 43 , 52 , 61 , 81 ); adapting to new conditions ( 49 , 54 , 61 , 68 , 79 ); improving decision making skills ( 47 , 50 , 52 , 72 , 80 ); crisis resolution skills ( 34 ); management skills ( 39 , 52 , 63 ); leadership skills ( 41 , 49 , 52 , 68 ); teaching skills ( 43 , 48 , 52 , 68 ); academic achievement ( 47 , 48 , 61 ); increasing a collaborative spirit ( 5 ); personal satisfaction ( 33 , 62 , 70 , 75 ); team building skills ( 40 , 45 , 50 , 59 , 65 ); career planning skills ( 59 , 66 , 67 ); negotiation skills ( 58 , 59 ); conflict resolution skills ( 59 ); teamwork skills ( 45 , 53 , 79 ); lifelong learning ( 47 , 53 , 63 ); sense of belonging ( 40 , 61 , 80 ); increasing academic productivity ( 14 , 58 , 59 ); developing social skills ( 2 , 33 , 49 ); helping students choose careers ( 33 ); increasing research productivity ( 33 ); raising awareness ( 33 ); growing professional identity ( 33 , 35 , 61 , 62 , 70 , 77 ); increasing well-being ( 33 , 62 , 66 , 68 ); student career planning ( 41 , 62 ); achieving personal goals ( 2 , 33 , 74 ); achieving academic goals ( 33 ); shaping academic personality ( 33 ); problem solving skills ( 40 , 49 , 50 , 72 ); academic satisfaction ( 64 , 72 ); active learning ( 43 , 50 , 55 , 58 ); effectiveness of educational activities ( 43 ); collaborative learning ( 39 , 45 , 58 ); increasing administrative skills ( 39 ); academic success ( 39 , 66 , 73 ); time management skills ( 38 , 60 , 77 ); increasing planning ability ( 39 ); learning new skills ( 36 , 39 ); session management skills ( 39 ); metacognition skills ( 41 , 79 ); developing students’ social responsibility ( 41 ); learning technical skills ( 81 ); achieving professional and academic competencies ( 50 , 81 ); developing specialized medical skills ( 34 , 54 , 80 ); developing creativity ( 48 , 73 , 79 ); improving analytical skills ( 64 ); analytical thinking ( 67 ); career counseling ( 64 ); managing academic pressure ( 64 ); supporting study skills ( 75 ); providing guidelines for success in medicine ( 75 ); increasing student motivation ( 43 , 50 , 65 ); promoting the quality of student articles and dissertations ( 73 ); increasing statistical analysis skills ( 73 ); increasing interest in field of study ( 42 ); self-efficacy ( 58 , 61 , 66 ); career advancement ( 34 , 54 , 70 ); career development ( 2 , 60 ); job satisfaction ( 67 , 74 , 78 ); self-consciousness ( 55 , 74 ); increasing self-esteem ( 38 , 49 , 70 , 77 ); feeling productive ( 67 ); research innovation ( 58 ); developing analytical skills ( 58 ); increased ability to deal with difficult situations ( 67 ); becoming professional ( 56 , 77 , 79 ); communication skills ( 49 , 50 , 54 , 68 ); practical skills ( 50 , 56 ); help with career guidance ( 54 , 56 , 68 ); career preparation ( 47 ); increased ability to recognize ( 47 ); increased interest in field of study ( 80 ); evaluating career option ( 68 ); obtaining more specialized information ( 68 ); increased student retention rate ( 35 , 36 , 40 , 65 ); cognitive growth ( 49 ); problem-based learning ( 38 ); acquiring skills needed for the future ( 56 ); **b) academic outcomes:** scholarly sociability ( 59 , 66 ); attracting potential future faculty ( 66 ); socializing the students ( 37 , 73 , 80 ); increasing graduation rate ( 41 ); training future physicians ( 41 , 64 ); increasing organizational commitment ( 75 ); preventing academic erosion ( 61 ); creating a learning community ( 48 ). The last component is the consequences of implementation a mentoring program. As the findings show, the implementation of a mentoring program has many benefits for student development. In addition, it has positive consequences for medical universities. In fact, medical students will have better academic performance when their various abilities improve. Also, they help improve the quality of health system performance in the future. Mentoring strategy is one of the best and least costly strategies for the development of medical students. The following Figure 2 shows the percentage of each component.

According to this chart, the highest rate is related to (Outcomes of program implementation) with 45%. It emphasizes the importance and necessity of implementing mentoring programs for medical students. Of course, in order to have a successful program, all the steps must be performed accurately. In other words, all the identified components indicate what components make up a successful mentoring program, and they are all equally important in terms of performance.

## Discussion

After analyzing and synthesizing the international studies, seven components were emerged in the field of student mentoring programs. The first component is academic. It includes leadership and management in the university. The important point is the participation of all stakeholders in decision making in the university. Besides, the development of performance-based reward and punishment systems that are about the actions and performance of academics can be a motivating factor for better implementation of mentoring programs for medical students. Furthermore, formal mentoring programs should be systematically addressed in university documents to align with the university’s mission and goals. The results of this component are consistent with that of the previous studies ( 33 , 39 , 50 , 52 - 54 , 58 , 60 , 62 , 63 , 66 , 67 , 70 , 72 , 73 , 81 ). The second component is communication. It aims to promote social capital to stabilize and connect intra- and inter-university relationships among academics in medical universities. The scope of this communication is vast and diversified, and it involves students as well as university management in order to more effectively conduct the mentorship program. Another issue is communication management, which is the establishment of a setting to foster and enhance communication. This finding is consistent with what has been found in communication studies ( 34 , 35 , 38 , 41 , 48 , 49 , 57 , 59 , 60 , 64 , 68 , 70 , 74 , 76 , 77 , 79 , 80). 

The third component is the soft capacities. It represents academic culture norms and moral norms. The foundation of any change is the academic culture and the subcultures that define it. Moreover, a kind of collectivism must prevail in academic culture to form a common identity and language among academics in medical universities. Moral norms refer to ethical standards and attitudes such as academic ethics, moral obligation, ethical behavior, etc. The findings of this component are in line with earlier research ( 33 , 36 , 39 , 41 , 42 , 44 , 47 , 48 , 52 , 53 , 58 , 65 , 71 , 72 , 77 , 79 ). Prior to program implementation, there is a fourth component. Prior to the execution of a program, it is critical to consider planning and preparation. Although a program may have been meticulously developed, it may not have been adequately organized or prepared prior to deployment. Such a scheme will fail miserably. However, it is important to prepare the key actors of the mentoring program (faculty and students). This can be done by conducting introductory workshops, preparing brochures and writing guidance on how to implement the program, and conducting a needs assessment to obtain the views of the key actors in the program to make the medical student mentoring program more effective. The results of this component are consistent with those of the previous studies ( 2 , 39 , 41 , 44 , 47 , 51 , 54 , 55 , 60 , 62 , 64 , 72 - 74 , 81).

The fifth component is the implementation time of the program. It indicates how the mentoring program is implemented and managed. During the implementation of the program, a schedule should be designed in defining the objectives, tasks, and plans to achieve the goals. Moreover, regular sessions, commitment and mutual responsibility to fully implement the sessions, and diversification of communication tools should be considered during the implementation of the program. Another significant consideration is the consistency of the substance and themes of the sessions, as well as the structuring of the program's execution with stated mentoring goals, which must be continually reviewed. This component's findings are consistent with earlier study findings ( 14 , 33 , 37 , 39 , 46 , 55 - 57 , 62 , 63 , 66 , 67 , 71 , 77 , 80 ). The sixth component is monitoring and evaluating the program implementation. In addition to effectively and seriously monitoring the program implementation, the results of program performance should be systematically reviewed and the results be regularly used to improve the program. It is important to pay attention to the positive outcomes and encouragement in various forms of promotion, salary, financial rewards, appreciation, etc. to further motivate the academics in order to improve and continuously update the program. The results of this component are consistent with the research findings ( 2 , 33 - 37 , 39 , 41 , 43 , 46 , 48 , 49 , 52 , 53 , 55 - 57 , 59 , 60 , 62 - 66 , 68 , 71 , 74 , 81 ). The last component is the outcomes of program implementation. The range of usefulness of the outcomes of program implementation can be very broad and varied as the pieces of the puzzle are put together in the previous steps. The results of this component, based on research findings, confirm this point. The results are divided into medical student outcomes (dominant proportion) and academic outcomes. Furthermore, the diversity of student interests implies that these programs are very successful in building medical students' human capital, which has good implications and advantages for medical universities. The results of this component are consistent with findings from previous studies ( 2 - 6 , 14 , 33 , 34 , 39 , 46 - 48 , 50 - 52 , 56 - 62 , 65 , 67 - 71 , 74 - 76 , 79 - 81 ).

## Conclusion

This article attempted to provide a more comprehensive vision of mentoring programs via the synthesis and analysis of studies. Mentoring programs for medical students have a systematic method, and we need to pay attention to all phases to implement them effectively. A benefit of such research is that it combines the results of a single study on mentoring medical students, so allowing for a more thorough grasp of the issue because its components have yet to be identified in the worldwide mentoring literature in the medical sciences. As a result, the unique contribution of such research is the identification of components at both the individual and university levels. The disadvantage of this research is the lack of using quantitative methods that can be effective as a complement to the qualitative method. Therefore, further research is suggested to study this issue in the University of Medical Sciences with mixed methods research. Researchers can evaluate the situation of Iranian medical universities by converting the components of this research into a questionnaire using advanced statistical methods such as multilevel analysis. Limitations of research: This research is qualitative and due to the nature of such research, it has little generalizability; another limitation of the Meta synthesis method is related to the gray literature. Other research in non-English languages on mentoring may not have been considered in this study. Finally, policy recommendations are offered for the health academic system:

◦ Raising the awareness of mentoring programs and their importance in upstream academic health care documents and in the strategic programs of medical universities;
◦ Sensitization of the medical university managers to the importance of these programs and the accurate design of all their phases, as well as a long-term capitalistic view of these programs;
◦ Changing subcultures at the medical department level to create a university culture that supports mentoring programs;
◦ Developing academic communication in medical universities to improve the social capital of the university and increase the effectiveness and efficiency of mentoring programs;
◦ Preparing and training medical faculty and students to best implement the program;
◦ Continuously monitoring program implementation along with reporting and receiving feedback on performance and using evaluation results to improve the program;
◦ Providing more funding for medical student mentoring programs in terms of the scope and diversity of their benefits.

## Authors' contribution

A.K, E.K contributed to the conception and design of the work; the acquisition, analysis, or interpretation of data for the work. All Authors contributed in drafting and revising the manuscript critically for important intellectual content. All authors have read and approved the final manuscript and agree to be accountable for all aspects of the work in ensuring that questions related to the accuracy or integrity of any part of the work are appropriately investigated and resolved.

## Conflict of Interest:

None declared.
